# Increased enzymatic hydrolysis of sugarcane bagasse from enzyme recycling

**DOI:** 10.1186/s13068-014-0185-8

**Published:** 2015-01-22

**Authors:** Evan Michael Visser, Tiago Ferreira Leal, Maíra Nicolau de Almeida, Valéria Monteze Guimarães

**Affiliations:** Departamento de Bioquímica e Biologia Molecular, Universidade Federal de Viçosa, Campus Universitário, 36570-000 Viçosa, MG Brazil

**Keywords:** Enzyme recycling, Cellulosic biofuels, Bioethanol, Glucose, Lignin

## Abstract

**Background:**

Development of efficient methods for production of renewable fuels from lignocellulosic biomass is necessary to maximize yields and reduce operating costs. One of the main challenges to industrial application of the lignocellulosic conversion process is the high costs of cellulolytic enzymes. Recycling of enzymes may present a potential solution to alleviate this problem. In the present study enzymes associated with the insoluble fraction were recycled after enzymatic hydrolysis of pretreated sugarcane bagasse, utilizing different processing conditions, enzyme loadings, and solid loadings.

**Results:**

It was found that the enzyme blend from *Chrysoporthe cubensis* and *Penicillium pinophilum* was efficient for enzymatic hydrolysis and that a significant portion of enzyme activity could be recovered upon recycling of the insoluble fraction. Enzyme productivity values (g glucose/mg enzyme protein) over all recycle periods were 2.4 and 3.7 for application of 15 and 30 FPU/g of glucan, representing an increase in excess of ten times that obtained in a batch process with the same enzyme blend and an even greater increase compared to commercial cellulase enzymes.

**Conclusions:**

Contrary to what may be expected, increasing lignin concentrations throughout the recycle period did not negatively influence hydrolysis efficiency, but conversion efficiencies continuously improved. Recycling of the entire insoluble solids fraction was sufficient for recycling of adhered enzymes together with biomass, indicative of an effective method to increase enzyme productivity.

## Background

The production of bioethanol from lignocellulosic biomass is a subject of great interest because cellulosic ethanol presents the potential to substitute gasoline, promote rural development, and reduce greenhouse gases, while utilizing material not fit for human consumption [1]. The hydrolysis process for conversion of cellulose to glucose can be either acid or enzyme catalyzed. Acid-catalyzed hydrolysis yields sugars from highly complex biomass, but requires either high temperatures or high acid concentrations which can often make the process economically infeasible [2]. However, the major bottleneck of enzyme-catalyzed hydrolysis is the high cost of enzymes and relatively low yields.

To overcome low hydrolysis efficiencies, multiple processing configurations have been assessed; however, often high energy costs and the use of concentrated chemicals complicate the process and may make it uneconomical [3,4]. Pretreatment methods generally seek to remove lignin and hemicellulose from the biomass, leaving cellulose, which is more readily hydrolyzed when free of the hemicellulose and lignin fractions. Utilization of hemicellulase-rich enzyme extracts may be a potential solution to the use of pretreatments seeking to hydrolyze the hemicellulose fraction via thermochemical methods, in which enzymatic hydrolysis would result in significantly fewer inhibitors.

The blending of enzyme extracts is a strategy used to improve enzyme hydrolysis [5,6]. Blending crude enzyme extracts from different fungi has received less attention than blending specific enzymes, but it shows great potential since no activities are lost in concentration/purification processes, and thus a wide spectrum of enzyme activities is maintained. Synergy among enzymes from individual enzyme extracts is also another advantage to utilization of these enzyme blends [7,8].

The low cellulose content of pretreated biomass also contributes to complicate the acquisition of high product concentrations. Lignin is typically viewed as one of the major inhibitors to enzymatic hydrolysis, and it also accumulates in fed-batch processes since it is not broken down by cellulase enzymes and therefore remains in an insoluble form [9]. Intense pretreatment methods are often utilized to reduce lignin concentrations; however, lignin degradation products include phenolic acids, particularly tannic and gallic acids, which inhibit cellulase enzymes. Washing the pretreated biomass with hot water improves enzyme digestibility since at least some of the inhibitors formed are water soluble [10]. Laccase enzymes have been utilized to oxidize phenols and therefore improve lignocellulolytic enzyme efficiency [11].

Fed-batch processes have been utilized in an attempt to improve product yields, which may suffer from high solids loadings in which the solid substrate is continuously or intermittently fed with the solid substrate. Strategies for using this process are typically categorized into three main groups: (i) enzyme recycling, (ii) fed-batch simultaneous saccharification and fermentation (SSF) processes for mitigation of the inhibitory effect caused by hydrolysis products, and (iii) increase of the cumulative substrate in a hydrolysis reactor [12]. These fed-batch systems have been applied for enzyme saccharification and sometimes fermentation of various different biomasses to increase final concentrations of sugars or ethanol [9,13,14].

The objective of the present study was to determine if recycling of the insoluble solids fraction could enable a significant portion of the enzyme activity to be reutilized, thus resulting in an increased overall yield or a decrease in the quantity of enzyme required for conversion. Two experiments were therefore performed to evaluate recycling of the solids fraction: one assessed successive additions of a predefined biomass quantity, and in the other biomass was added in order to maintain a determined solids loading. An enzyme blend consisting of extracts from the fungi *Chrysoporthe cubensis* and *Penicillium pinophilum* was used which previously showed excellent lignocellulose hydrolysis potential and synergistic action. Different enzyme loadings were assessed to evaluate the effect of the recycled enzymes adhered to the solids fraction. The influence of the recycled lignin-rich residue was also assessed with respect to its effect on enzyme hydrolysis.

## Results and discussion

### Characterization of the biomass and enzyme blend

Sugarcane bagasse was utilized as a model substrate for the saccharification experiments due to its availability as a potential lignocellulosic feedstock in Brazil. Prior to enzymatic hydrolysis, the bagasse was submitted to thermochemical pretreatment with 1.5% NaOH at 120°C for one hour after which approximately 50% of the lignin was removed (Table 1), thus facilitating enzyme attack of the cellulose and hemicellulose fractions.

**Table 1:** **Composition of the raw and pretreated sugarcane bagasse**

**Biomass component**	**Untreated bagasse (%)**	**Pretreated bagasse (%)**
Glucan	52.8	59.2
Xylan	19.1	22.3
Arabinan	1.6	2.1
Lignin	22.1	11.4

Values are the average of three repetitions; standard deviations did not exceed < 10% of the mean.

The enzyme cocktail utilized in the saccharification assays with enzyme recycling was the same as that developed in a previous study evaluating synergism between enzyme extracts produced by the fungi *Chrysoporthe cubensis* and *Penicillium pinophilum* [8]*.* In the cited study it was found that synergy of the FPase and endoglucanase activities among the two extracts was 76% and 48% greater than theoretical values, respectively. The concentrated enzyme blend utilized in the present study presented activities of 5.7 FPU/mL, 32.3 U/mL, 23.21 U/mL, and 176.95 U/mL for FPase, endoglucanase, xylanase, and β-glucosidase activities, respectively. The total enzyme protein determined according to the Coomassie Blue binding method was 2.2 mg/mL. From previous publications on this same enzyme blend as well as enzyme production from *Chrysoporthe cubensis*, it is clear that an array of enzyme activities are present in the blend utilized [8,15].

### Ability of recycled cellulase and xylanase enzymes to hydrolyze freshly added biomass

**Figure 1:**
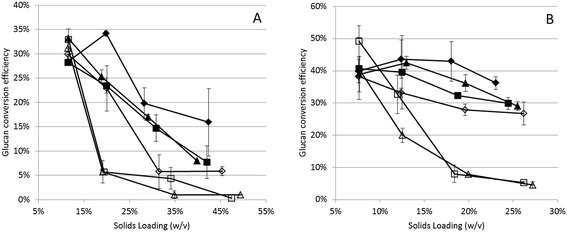
**Conversion efficiencies of fresh glucan as a function of biomass loading for treatments receiving an additional 12% (A) and 8% (B) solids loading at each hydrolysis cycle.** In figure **A** the treatments are represented as: 1 (solid diamonds), 3 (solid squares), 5 (solid triangles), 7 (open diamonds), 9 (open squares), and 11 (open triangles). In figure **B** the treatments are represented as: 2 (solid diamonds), 4 (solid squares), 6 (solid triangles), 8 (open diamonds), 10 (open squares), and 12 (open triangles), according to Table 2.

Based on the amount of glucose and xylose produced during the course of the saccharification reactions, it was observed that significant quantities of these sugars were produced from both the fresh and residual substrates when insoluble solids were recycled. Figure 1 indicates that although the overall fresh glucan conversion decreased as a function of increasing solids loading, in some cases the conversion efficiency increased in the second hydrolysis period resulting from recycled enzyme activity with the solids fraction. As expected, this increase in conversion efficiency is best observed in the treatments receiving greater enzyme loadings upon recycling and for the lower solids loading (Figure 1B). The treatments in which no fresh biomass was added are not included in Figure 1, since there was no biomass accumulation.

As has been observed in other diverse studies, increased solids loading negatively affected enzyme hydrolysis efficiency [16]. Treatments receiving additional biomass at the concentration of 12% (Figure 1A) showed a much more rapid decline in glucose efficiency than the treatments receiving 8% biomass (Figure 1B). It was also visually verified that when 25% solids was exceeded, the slurry was paste-like, which limits enzyme mobility [17]. Because the reaction vessel was submitted to agitation without direct contact between the agitation source and the biomass slurry, enzyme access to the biomass was likely limited if the biomass slurry was not properly mixed.

**Figure 2:**
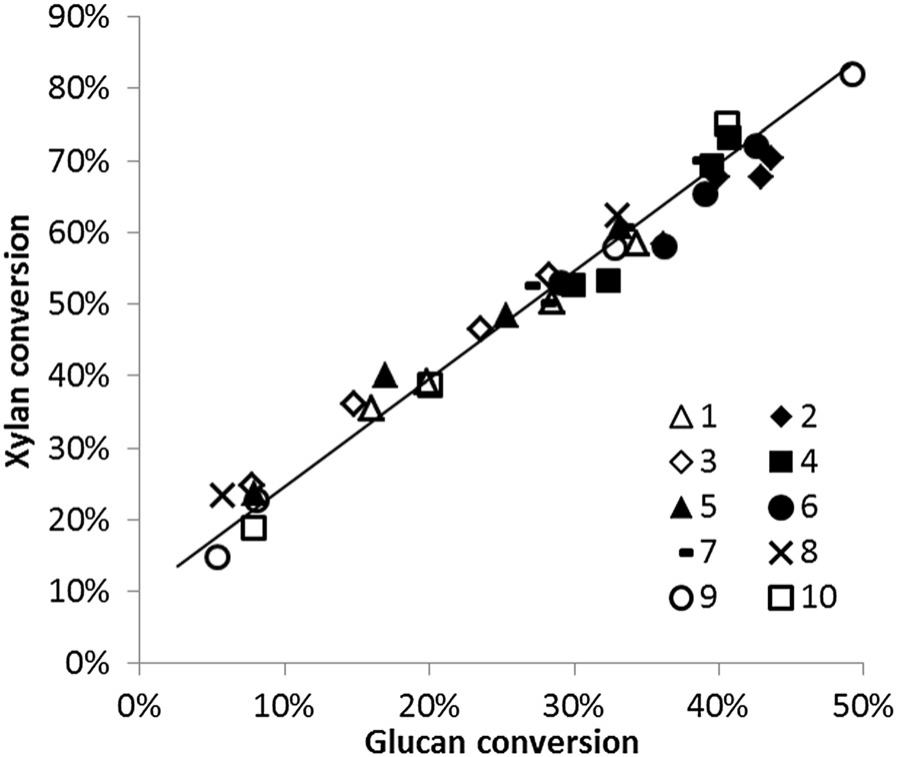
**Relationship between glucan and xylan conversion to their respective monosaccharides.** Points reflect the diverse recycle numbers for the different treatments (see Table 2).

The xylan conversion efficiency was always greater than that of glucan, showing a linear correlation (Figure 2). This is surely due to the amorphous structure of hemicellulose, its lower degree of polymerization [18], and the high xylanase activity of the enzyme blend, and was similar to the results shown when evaluating the effect of supplementing hemicellulases for saccharification of lignocellulosic biomass [5]. Therefore, the efficiency of the xylan hydrolysis process appears to directly affect the glucan hydrolysis efficiency, indicating the importance of removing xylan from the biomass structure to maximize glucose yields. The enzyme extract produced by *Chrysoporthe cubensis* has previously been shown to be an excellent source of xylanase enzymes, as well as additional accessory enzymes that aid in the biomass hydrolysis process [15]. Synergistic enzyme action was also confirmed when blending the *C. cubensis* extract with that of *P. pinophilum*, where *P. pinophilum* produces greater quantities of cellulase enzymes [8]. This is one of the advantages of blending crude enzyme extracts. No accessory enzymes are lost in purification steps, which may result in synergistic effects [19].

**Figure 3:**
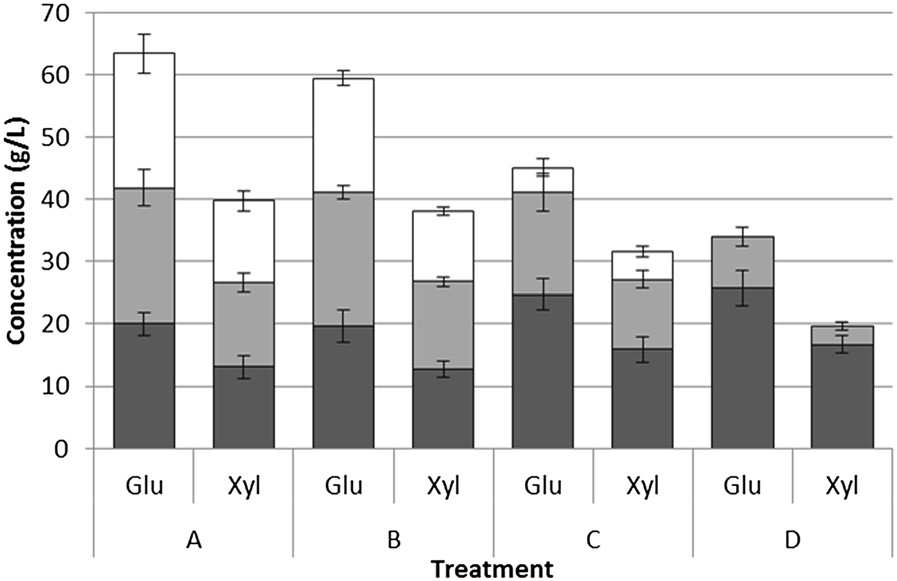
**Masses of glucose and xylose produced in treatments 2, 6, 10, and 14, according to Table** **2** **, represented by the letters A, B, C, and D.** Bottom bars (black) represent the glucose and xylose produced in the first hydrolysis period, the middle bars (gray) are the masses of sugars produced in the second hydrolysis period, and the top bars (white) represent the sugar masses produced in the third hydrolysis period. Error bars represent ± one standard deviation.

To best observe the effects of recycled enzyme activity, the four different enzyme loading treatments were compared for the same biomass loading, where the highest initial enzyme loading (20 FPU/g) and lowest biomass loading (8%) best compared the results (see Figure 3 showing treatments 2, 6, 10, and 14 according to Table 2). In the second and third round of hydrolysis both glucose and xylose were produced in all treatments receiving additional biomass, independent of the additional enzyme loading (1X, 1/2X or 0). The fact that glucose and xylose were continuously liberated even when no fresh enzyme extract was added indicated recycling of enzyme activity; even more remarkable was the fact that sugar liberation was nearly identical in the treatment receiving only half of the original enzyme loading upon recycling compared to that receiving the full enzyme loading. In the treatment receiving the fresh enzyme extract with no additional biomass (Figure 3D), the reduced liberation of hydrolysis products is more due to substrate availability than lack of the enzyme catalyst [20].

**Table 2:** **Experimental assays for analyzing the effect of insoluble solids recycling**

	**Treatment**															
	**1**	**2**	**3**	**4**	**5**	**6**	**7**	**8**	**9**	**10**	**11**	**12**	**13**	**14**	**15**	**16**
Enzyme loading 1 (FPU/g)	20	20	10	10	20	20	10	10	20	20	10	10	20	20	10	10
Solids loading 1 (% w/v)	12%	8%	12%	8%	12%	8%	12%	8%	12%	8%	12%	8%	12%	8%	12%	8%
Enzyme loading 2 (FPU/g)	20	20	10	10	10	10	5	5	0	0	0	0	20	20	10	10
Solids loading 2 (% w/v)	12%	8%	12%	8%	12%	8%	12%	8%	12%	8%	12%	8%	0%	0%	0%	0%
Enzyme loading 3 (FPU/g)	20	20	10	10	10	10	5	5	0	0	0	0	20	20	10	10
Solids loading 3 (% w/v)	12%	8%	12%	8%	12%	8%	12%	8%	12%	8%	12%	8%	0%	0%	0%	0%
Enzyme loading 4 (FPU/g)	20	20	10	10	10	10	5	5	0	0	0	0	20	20	10	10
Solids loading 4 (% w/v)	12%	8%	12%	8p%	12%	8%	12%	8%	12%	8%	12%	8%	0%	0%	0%	0%

Recycling of the lignocellulolytic enzymes occurs because the enzymes readily adsorb to the solid substrates. Because the enzymes β-glucosidase and β-xylosidase have soluble substrates (cellobiose and xylobiose), there are conflicting reports on whether they absorb to solid lignin [21-23]. In the present experiment no accumulation of cellobiose was observed, even in the assays receiving no fresh enzymes upon recycle, indicating that β-glucosidase was potentially bound to the solid fraction. Cellulase adsorption by lignin has also been reported when assessing enzymatic hydrolysis, further preventing enzymes from being removed with the liquid fraction [24,25]. Additional information on the effects of cellulase adsorption by lignin is presented in the section entitled Effect of increased lignin concentrations on enzymatic hydrolysis.

### Fixed solids concentration

Having observed the potential for recycling of enzymes together with insoluble solids, as well as the negative influence of excessive biomass concentrations, we opted to perform a hydrolysis test with constant product monitoring which permitted us to estimate the percentage of biomass hydrolyzed and therefore the biomass concentration. By maintaining a consistent biomass loading it was expected that a significantly higher enzyme productivity (quantity of sugar produced per quantity of enzyme applied) would be achieved since mixing will not be significantly affected by overwhelming solids concentrations. In this experiment a magnetic agitation bar was used for direct agitation of the biomass slurry.

In this experiment it was desired to maintain a solids loading of 12%; thus, at 48-h intervals biomass was added to the hydrolysis medium to maintain this concentration. At 96-h intervals additional volumes of the enzyme cocktail were added together with fresh biomass, at concentrations of 15 or 30 FPU per gram of glucan (7 or 14 mg enzyme protein per gram of glucan). Enzyme loading was defined as a function of glucan mass instead of total biomass due to the ever-changing composition of the solid fraction. By maintaining a constant solids loading, proper mechanical agitation could be maintained so as not to inhibit enzyme activity. However, as in any recycle loop, there is a potential for buildup of contaminants, and in this case lignin accumulation is expected. Maintaining a constant solid fraction is also more attractive from an industrial viewpoint since it allows for greater process control.

**Figure 4:**
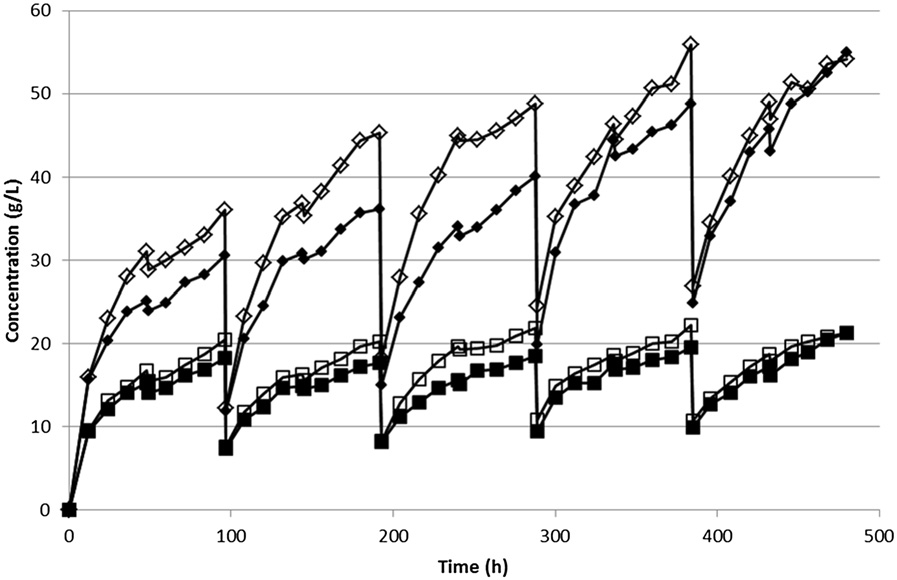
**Production profile of glucose and xylose for the two enzyme loadings.** The 30 FPU/g treatment is represented by open symbols and the 15 FPU/g treatment by filled symbols (diamonds indicate glucose and squares indicate xylose). Relative standard deviations between measurements were less than 5% in all cases.

The results of recycling the entire insoluble solids fraction showed increased sugar concentrations (Figure 4). Large decreases in sugar concentration were observed at 96-h intervals when the hydrolysis mixture was centrifuged and the sugar-containing supernatant was removed, followed by addition of the fresh enzyme blend, buffer, and biomass, which diluted the residual sugar content. The small decreases in sugar concentration at 48, 144, 240, 336 and 432 h were the result of the biomass addition, where the biomass contained 60% water and thus resulted in a small dilution of the hydrolysis medium. Because total sugar liberation was nearly the same during each of the recycle periods, it may be suggested that biomass composition did not affect enzyme hydrolysis. However, the slower release of sugars during the period in which additional biomass was added without supplementing enzymes may be indicative of partial inhibition by the product (sugars).

Cellobiose did not accumulate in either of the experiments. Because the substrate for the enzyme β-glucosidase is soluble (cellobiose), if linked to this substrate it would be lost with the liquid fraction. It is possible that β-glucosidase activity was encountered in multienzyme complexes which have been observed for different fungi [26,27]. However, β-glucosidase has also been found to strongly bind to lignin-rich residues [28]. Cellulase binding to lignin has commonly been thought to be one of the major forms of enzyme inhibition in the process of converting lignocellulose to soluble sugars [29]. In a study evaluating cellulase adsorption by lignin, cellulase adsorption was maximized in the first hour when glucan hydrolysis rates were maximized, followed by decreasing cellulase adsorption [30].

**Figure 5:**
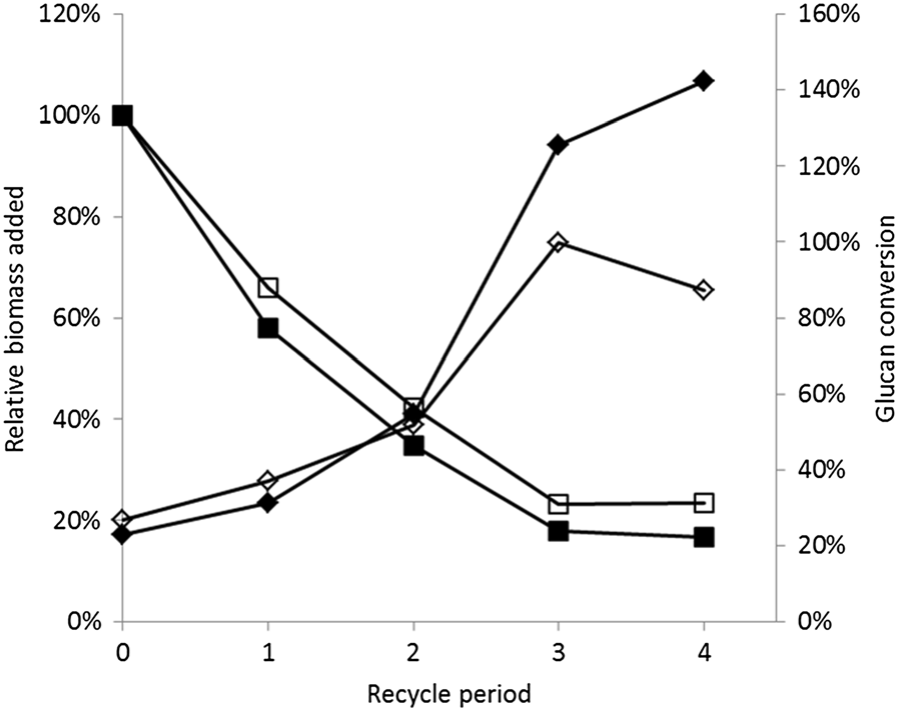
**Glucan conversion efficiencies (diamonds) and relative biomass added per recycle period (squares).** Filled shapes denote the enzyme loading of 15 FPU/g glucan and open shapes denote the enzyme loading of 30 FPU/g glucan.

Glucose conversion increased as recycling increased for both enzyme loadings (Figure 5). As of the third recycle period, conversions of the fresh glucan equivalents were 100% for the higher enzyme loading (30 FPU/g) and 125% for the lower enzyme loading (15 FPU/g), indicating that total glucan conversions were equivalent to conversion of all fresh glucan added and all fresh glucan plus a portion of recycled glucan, respectively. A small decrease in glucan conversion efficiency was observed in the last recycle period for the treatment receiving 30 FPU/g glucan; however, the conversion of fresh glucan added was still approximately 90%. This slight decrease in conversion efficiency may be related to excessive buildup of contaminants from the multiple recycles. The reduced glucose liberation in the treatment receiving the higher enzyme loading may be due to the greater glucan concentration in the biomass submitted to the 15 FPU treatment (52%) compared with the 30 FPU treatment (48%).

### Comparison of batch and fed-batch hydrolysis

**Figure 6:**
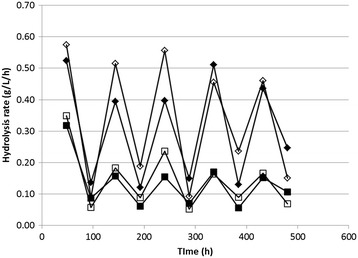
**Rates of glucose and xylose liberation for the two enzyme loadings.** Glucose liberation rates are represented by diamonds and xylose liberation rates are represented by squares (filled shapes denote the enzyme loading of 15 FPU/g glucan and open shapes denote the enzyme loading of 30 FPU/g glucan).

A previous study was performed utilizing the same enzyme blend on alkali-pretreated sugarcane bagasse in a 120-h batch hydrolysis [8]. In that study it was observed that the hydrolysis rate decreased to less than 0.2 g/L/h after just 60 h for the varying biomass and enzyme loadings. In the present study the hydrolysis rate was maintained higher, as observed in Figure 6, which shows the glucose and xylose liberation rates over 48-h intervals. Over the full 488-h test period, the average glucose liberation rates were 0.31 and 0.33 g/L/h for the treatments with 15 FPU/g glucan and 30 FPU/g glucan, respectively, and xylose was liberated at rates of 0.15 and 0.16 g/L/h. The proximity of these values indicated that extending the hydrolysis period would be more effective than increasing the enzyme loading, since when utilizing only half the enzyme loading the glucose liberation rate was only 6% lower.

Enzyme productivity (quantity of sugar produced per quantity of enzyme applied) is one of the best methods for comparison of enzyme efficiency. This parameter was utilized in another enzyme recycling study when applying a commercial enzyme extract, with significantly higher enzyme loadings [21]. The study performed by these authors presented maximum enzyme productivity in the range of 0.3 g glucose/mg enzyme protein (Table 3). In the present study this number was drastically higher, where the greatest productivity was obtained when applying 15 FPU/g of glucan (3.74 g glucose/mg enzyme protein). When using this same enzyme cocktail in a batch experiment, a maximum productivity of 0.37 g glucose/mg enzyme protein was obtained, indicating the efficiency of the fed-batch process in significantly increasing productivity [8]. The high productivity values obtained are likely due to the complex nature of the enzyme extract utilized. Inclusion of hemicellulase enzymes in the extract has been shown to greatly improve enzymatic hydrolysis of both xylan and cellulose, without the need for exceptionally high cellulase loadings [5,31].

**Table 3:** **Results of fed-batch processing when maintaining a constant solids loading (12% w/v) and varying the enzyme loading**

	**15 FPU/g cellulose**		**30 FPU/g cellulose**	
	**Glucose**	**Xylose**	**Glucose**	**Xylose**
Average rate (g/L/h)	0.31	0.15	0.35	0.16
Overall conversion efficiency	51%	64%	55%	68%
Enzyme productivity (g sugar/mg enzyme protein)	3.78	1.79	2.56	1.19

### Effect of increased lignin concentrations on enzymatic hydrolysis

**Figure 7:**
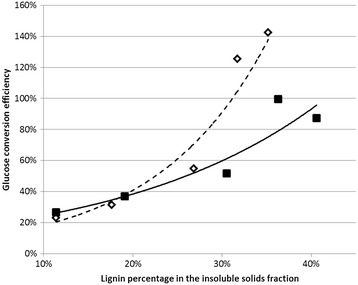
**Theoretical glucose yields for the 96-h hydrolysis reactions on the pretreated sugarcane bagasse.** Open diamonds, dashed tendency line, represent the enzyme loading of 15 FPU/g glucan; filled squares, solid tendency line, denote the enzyme loading of 30 FPU/g glucan.

It was found that in the treatments with enzyme loadings of both 15 and 30 FPU/g, hydrolysis efficiency increased with increasing lignin concentrations. Because the entire soluble solids fraction was reutilized in all recycle periods, it was expected that lignin would build up in the hydrolysis medium, since it is not efficiently broken down and solubilized as occurs with the cellulose and hemicellulose fractions. This can be observed when comparing the lignin percentage to the efficiency for converting fresh glucan added to the reaction medium (Figure 7). The results differ significantly from what is expected, because lignin is known to nonspecifically bind to cellulase enzymes and therefore inhibit cellulose hydrolysis [22].

Phenolic hydroxyl groups associated with lignin particles are able to adsorb proteins and deactivate cellulolytic enzymes [32]. Further assessment of the enzyme extract indicated the presence of laccase activity equivalent to 2.4 U/mL. Laccase enzymes are capable of oxidizing phenols, and their presence may further assist in preventing cellulolytic enzyme deactivation [11].

Adsorption of enzymes to the substrate during hydrolysis is desired because productive binding of proteins on the substrate surface has been shown to improve enzyme-substrate interactions. However, non-productive binding of enzymes to lignocellulosic substrates also occurs, where the enzymes may remain blocked in the dead ends of the substrate or non-productively bind to either cellulose or lignin [33]. Besides non-productive binding, lignin may cause inaccessibility of cellulase enzymes to its substrate. Pretreatment methods also have different effects on lignin properties. Lignin in eucalyptus wood chips subjected to steam-explosion pretreatment showed a greater capacity to adsorb cellulase enzymes than non-pretreated lignin [34]. It has been shown that cellulase adsorption decreased over a 72-h hydrolysis period [30], and in the present study in which the hydrolysis period was significantly longer (a total of 488 h), this effect may have been extrapolated.

This same behavior of lignin was observed in another study evaluating the recycling of insoluble solids [21]. These authors suggested that because the lignin residue was previously exposed to the enzyme blend, the binding sites on lignin may have already been occupied by enzymes from the prior hydrolysis. It has already been shown that enzymes remain active when associated with the insoluble fraction, adhering to the cellulose and hemicellulose fractions as well as lignin, and it may also be possible that inactivated enzymes remain adhered to the solids and occupy lignin-protein binding locations. Visual assessment of the solid fraction after the multiple recycle periods indicated that the biomass, which initially was fibrous, presented a very fine texture. This homogeneity and reduced size of solids may have contributed to facilitate enzymatic hydrolysis although the solids concentration was maintained. The results shown in the present study therefore indicate that lignin in the insoluble fraction has no negative effect on glucose and xylose yields under the experimental conditions used herein.

## Conclusion

It was possible to efficiently recycle a significant portion of enzymes from the blend consisting of enzyme extracts from *Chrysoporthe cubensis* and *Penicillium pinophilum* by recycling the solid fraction after enzymatic hydrolysis of pretreated sugarcane bagasse. The glucose yields remained the same after two hydrolysis periods when biomass was constantly added at the same rate when comparing the treatments receiving the same initial loading, half the initial loading, and no additional enzyme. Although the efficiencies did decrease when using this method, it was clearly shown that the enzymes could be efficiently recycled.

When basing process control on maintaining a defined solids loading, it was found that greater soluble sugar yields could be obtained even when applying fewer enzymes. This represents a significant improvement to the lignocellulosic biomass process as well as reduction in required enzyme loading for hydrolysis. However, when recycling the solid fraction, the continued buildup of lignin affects the processing parameters. In the present study lignin concentrations exceeded 40%, but apparently had no negative effect on enzymatic hydrolysis since conversion efficiencies continued to increase throughout the recycling period. Enzyme recycling therefore shows potential to reduce enzyme requirements and operating costs for the production of bioethanol.

## Material and methods

### Biomass pretreatment and compositional analysis

Sugarcane bagasse, from which the sugars had already been extracted, was obtained from the Center for Research and Breeding of Sugarcane of the Federal University of Viçosa, Brazil. In the laboratory this bagasse was again washed and dried in an oven at 70°C until it reached a constant mass, after which it was further milled (particle size less than 1 mm) in a knife mill (Marconi, Piracicaba, SP, Brazil) and submitted to alkaline pretreatment prior to being employed in saccharification experiments. Sodium hydroxide 1.5% was used to pretreat the milled sugarcane bagasse samples at a solids loading of 10% (w/v); and treatments were performed in an autoclave at 120°C for 60 min. The pretreated materials were separated into solid and liquid fractions using a Buchner funnel fitted with filter paper. The solid fraction was washed thoroughly with distilled water, sealed in a hermetic vessel to retain moisture, and stored at -20°C.

The chemical composition of the untreated and alkali-treated sugarcane bagasse samples was determined using a modified Klason lignin method derived from the TAPPI Standard Method T222 om-98 [35] (Table 2). Extractive-free biomass (0.3 g) was incubated at 30°C with 3 mL of 72% H_2_SO_4_ for 1 h with occasional mixing. The slurry was then transferred into a penicillin bottle containing 84 mL of deionized water, and the flask was sealed with a rubber stopper and an aluminum seal. The bottle was placed in an autoclave calibrated at 118°C for 1 h, then the slurry was filtered through a medium coarseness sintered glass filter for gravimetric determination of acid-insoluble lignin. Concentrations of biomass sugars (arabinose, galactose, glucose, xylose, and mannose) in the filtrate were quantified using high performance liquid chromatography (HPLC), while acid-soluble lignin was determined by absorption measurements at 205 nm [36]. The HPLC system Dionex DX-300 (Dionex Co., Sunnyvale, CA, USA) was equipped with a Carbopac PA1 column and a pulsed amperometric detector with a gold electrode. Prior to injection, the samples were filtered through 0.45-mm HV filters, and a volume of 20 μL was loaded into the chromatograph system. The column was pre-equilibrated with a NaOH solution, 300 mM, and elution was carried out at a flow rate of 1.0 mL/min at room temperature.

### Production of the enzyme blend

The enzyme extract utilized in the hydrolysis experiments was a 50:50 (v:v) blend of enzyme extracts from the filamentous fungi *Chrysoporthe cubensis* and *Penicillium pinophilum*. These fungi were cultivated according to the conditions stated in a previous study reporting the synergy of this enzyme blend and its potential for application in sugarcane bagasse hydrolysis [8]. The obtained enzyme blend was concentrated using a Micron ultrafiltration unit (Millipore Corporation, Bedford, MA) for later application in sugarcane hydrolysis assays. FPase, endoglucanase, β-glucosidase, and xylanase activities were measured according to previously published methods [35], and are presented in the results section.

### Enzyme assays

All enzymatic assays were carried out in sodium acetate buffer, 100 mM, pH 5, at 50°C, and in triplicate; the mean values were calculated and reported. The relative standard deviations of the measurements were below 5%. FPase and endoglucanase activities were determined using Whatman Grade 1 filter paper and carboxymethylcellulose as substrates, respectively, according to previously described standard conditions [37]. The total reducing sugar liberated during the enzymatic assays was quantified using the dinitrosalicylic acid (DNS) reagent [38] with glucose as a standard. Xylanase activity was determined using xylan from birchwood (1% w/v at final concentration). The enzymatic reactions were initiated by the addition of 100 μL of the appropriately diluted enzyme solution to 400 μL of the polysaccharide substrate solution prepared in buffer. The reaction mixtures were incubated for 30 min, and the total reducing sugar released was determined with the DNS reagent using xylose.

The β-glucosidase activity was measured using ρPNGlc as a substrate. The reaction mixtures contained 100 μL of the appropriately diluted enzyme solution, 125 μL of the synthetic substrate solution (1 mM at final concentration), and 275 μL of buffer. The reaction mixture was incubated for 30 min and stopped by addition of 0.5 mL sodium carbonate solution (0.5 M). The absorbance was measured at 410 nm, and the amount of ρ-nitrophenol released was estimated by a standard curve. One enzyme unit (U) is defined as the amount of the enzyme that catalyzes the conversion of one micromole of substrate per minute

The laccase enzyme activity was determined using 2,2´-azinobis(3-ethylbenzothiazoline-6-sulfonic acid) (ABTS) as a substrate. The reaction mixtures contained 100 μL of the appropriately diluted enzyme solution, 50 μL of the substrate ABST 10 mM, and 350 μL of buffer. These reaction mixtures were incubated for 10 min, and oxidation of ABTS was monitored by measuring absorbance at 420 nm. One enzyme unit was defined as the amount of the laccase that oxidized 1 μmol of the ABTS substrate per min.

### Protein determination

Protein concentration in the enzymatic extracts was determined by the Coomassie Blue binding method using bovine serum albumin as the standard [39].

### Enzyme hydrolysis experiments

Two sets of enzymatic hydrolysis experiments were performed; the first sought to assess the viability of recycling insoluble solids in sequential hydrolysis assays, and the objective of the second was to simulate a potential scenario for industrial application. These two experiments were referred to as those of “Increasing biomass concentrations” and “Consistent biomass concentrations.”

#### Increasing biomass concentrations

Enzymatic hydrolysis was performed in 2-mL micro-tubes with an initial working volume of 1 mL. The hydrolysis medium consisted of sugarcane bagasse, previously submitted to alkali-pretreatment and washing, at a concentration of 8% or 12% (w/v), distilled water, sodium acetate buffer pH 5 (final concentration of 50 mM), enzyme blend (20 FPU/g biomass), tetracycline (40 mg/L), and sodium azide (10 mM). The prepared micro-tubes were sealed and mounted horizontally in an orbital shaker at 200 rpm and 50°C.

At 96-h intervals the tubes were temporarily removed from the shaker and submitted to centrifugation at 8,000 *g* for 5 min, using an Eppendorf Centrifuge 5424. After this period the supernatant was transferred to a separate tube for sugar analysis. To the tube containing residual biomass the solution with the appropriate enzyme loading (see Table 2) was added to equally replace the removed supernatant fraction, and the same predetermined quantity of biomass was included so that the hydrolysis could be repeated. In the second round the hydrolysis conditions were modified to evaluate the addition of the same enzyme loading, addition of half the original enzyme loading, no additional enzyme loading, and addition of the original enzyme loading but with no additional biomass. The complete set of reaction conditions for the sequential hydrolysis assays is shown in Table 2.

#### Consistent biomass concentrations

Enzymatic hydrolysis was performed in 50-mL Erlenmeyer flasks with a working volume of 12 mL, which were then submitted to mechanical agitation. The hydrolysis medium consisted of alkali-pretreated biomass at a concentration of 12% (w/v), distilled water, sodium acetate buffer pH 5 (final concentration of 50 mM), enzyme blend (15 or 30 FPU/g glucan), tetracycline (40 mg/L), and sodium azide (10 mM). The prepared flasks were placed in a water bath at 50°C set upon magnetic agitators at 200 rpm.

The sugar content of the hydrolysis medium was monitored at 12-h intervals by acquiring samples of the liquid-solid mixture. The percentage of biomass converted to a soluble form was estimated based on the quantity of sugar liberated. At 48-h intervals additional biomass was added to the hydrolysis medium to maintain a solids loading of 10%. At 96-h intervals the hydrolysis medium was centrifuged at 8,000 x *g* (using a Beckman J2-MI centrifuge), the supernatant was removed for analysis, and the solids fraction was reloaded into the Erlenmeyer flasks along with the calculated fresh biomass, water, enzyme blend (15 or 30 FPU/g glucan), buffer, tetracycline, and sodium azide. This procedure was repeated four times over a period of 480 h.

### Hydrolysis product analysis

Products of the saccharification assays were analyzed by high performance liquid chromatography (HPLC) with a Shimadzu series 10A chromatograph. The HPLC was equipped with an Aminex HPX-87P column (300 x 7.8 mm, BioRad, Hercules, CA, USA) and a refractive index detector (Shimadzu Corporation, Kyoto, Japan). The column was eluted with water at a flow rate of 0.6 mL/min at 80°C.

## Abbreviations

ABTS: 2,2´-azinobis(3-ethylbenzothiazoline-6-sulfonic acid)
DNS: dinitrosalicylic acid
FPU: filter paper units
HPLC: high performance liquid chromatography
SSF: simultaneous saccharification and fermentation
U: enzyme unit
